# Novel polymorphisms and genetic studies of the shadow of prion protein gene (*SPRN*) in pheasants

**DOI:** 10.3389/fvets.2024.1399548

**Published:** 2024-05-14

**Authors:** Da-In Choi, Mohammed Zayed, Yong-Chan Kim, Byung-Hoon Jeong

**Affiliations:** ^1^Korea Zoonosis Research Institute, Jeonbuk National University, Iksan, Republic of Korea; ^2^Department of Bioactive Material Sciences and Institute for Molecular Biology and Genetics, Jeonbuk National University, Jeonju, Republic of Korea; ^3^Department of Surgery, College of Veterinary Medicine, South Valley University, Qena, Egypt; ^4^Department of Biological Sciences, Andong National University, Andong, Republic of Korea

**Keywords:** pheasants, prion, *SPRN*, polymorphism, SNP

## Abstract

**Background:**

Prion diseases in mammals are caused by the structural conversion of the natural prion protein (PrP^C^) to a pathogenic isoform, the “scrapie form of prion protein (PrP^Sc^).” Several studies reported that the shadow of prion protein (Sho), encoded by the shadow of prion protein gene (*SPRN*), is involved in prion disease development by accelerating the conformational conversion of PrP^C^ to PrP^Sc^. Until now, genetic polymorphisms of the *SPRN* gene and the protein structure of Sho related to fragility to prion disease have not been investigated in pheasants, which are a species of poultry.

**Methods:**

Here, we identified the *SPRN* gene sequence by polymerase chain reaction (PCR) and compared the *SPRN* gene and Sho protein sequences among various prion disease-susceptible and -resistant species to identify the distinctive genetic features of pheasant Sho using Clustal Omega. In addition, we investigated genetic polymorphisms of the *SPRN* gene in pheasants and analyzed genotype, allele, and haplotype frequencies, as well as linkage disequilibrium among the genetic polymorphisms. Furthermore, we used *in silico* programs, namely Mutpred2, MUpro and AMYCO, to investigate the effect of non-synonymous single nucleotide polymorphisms (SNPs). Finally, the predicted secondary and tertiary structures of Sho proteins from various species were analyzed by Alphafold2.

**Results:**

In the present study, we reported pheasant *SPRN* gene sequences for the first time and identified a total of 14 novel SNPs, including 7 non-synonymous and 4 synonymous SNPs. In addition, the pheasant Sho protein sequence showed 100% identity with the chicken Sho protein sequence. Furthermore, amino acid substitutions were predicted to affect the hydrogen bond distribution in the 3D structure of the pheasant Sho protein.

**Conclusion:**

To the best of our knowledge, this is the first report of the genetic and structural features of the pheasant *SPRN* gene.

## Introduction

Prion diseases are fatal, infectious neurodegenerative disorders caused by the misfolding of a benign prion protein (PrP^C^) into an abnormal prion protein (PrP^Sc^) (1). The exact process of converting PrP^C^ to PrP^Sc^ remains unclear, but several factors that play crucial roles in the conversion process have been identified (1, 2). The ability to infect some species and not others is a remarkable prion characteristic (3). A wide variety of species, including various members of mammalian families such as cattle, goats, deer, cats and other primate families, are susceptible to prion diseases (4–8). However, horses, dogs, and chickens are resistant to prion diseases (9–11). Previous studies have also indicated that genetic characteristics of the *SPRN* gene are significantly different between prion disease-susceptible species (cattle, goats) (12, 13) and -resistant species (horses, dogs, and chickens) (9, 10, 14). Therefore, the investigation of the prion disease-related genes in prion-resistant species has been of particular interest as it provides insights into the determinants of susceptibility.

Among the prion protein family, there are members such as shadow of prion protein (Sho) and PrP^C,^ which are mostly expressed in brain tissue (15, 16). These proteins have been reported to affect embryonic and mammary development (17). Previous studies suggest that the Sho protein, encoded by the shadow of prion protein gene (*SPRN*), interacts with PrP^C^ (18). This interaction speeds up the conversion of PrP^C^ to PrP^Sc^ (19). Since these two proteins interact and share similar characteristics, including protein expression profile and function (20, 21), it is essential to investigate the genetic characteristics of the *SPRN* gene to interpret the pathomechanism of prion diseases.

In the phylogenetic tree of the order Galliformes, chickens and pheasants have a close evolutionary relationship (22–24). Pheasants are members the genus *Phasianus* within the bird family (25). To date, prion infection has not been reported in birds and some other vertebrate species (26, 27). In our previous study, we reported 34 polymorphisms in the pheasant prion protein gene (*PRNP*), including 8 non-synonymous single nucleotide polymorphisms (SNPs) and 6 insertion/deletion polymorphisms (28). Furthermore, it has been reported that the pheasant PrP^C^ has a 3D structure similar to the chicken PrP^C^; thus, pheasant PrP^C^ is predicted to have relatively prion-resistant feature (28). Genetic polymorphisms in prion-related genes contribute significantly to interindividual variation, so they have been investigated as useful biomarkers in medicine, as well as in the study of pathology, pharmacology, epidemiology, and clinical immunology (29). In addition, polymorphisms of the *SPRN* gene in prion disease-susceptible species play a crucial role in determining their susceptibility to prion diseases (12, 30, 31). The *SPRN* polymorphisms, which contribute to expression level or protein function, show strong associations with the pathogenesis of prion diseases in different species (32, 33). However, the *SPRN* gene sequence, genetic polymorphisms, and structural features of Sho in pheasants have not yet been investigated.

In this study, we amplified the *SPRN* gene sequence by polymerase chain reaction (PCR) and performed multiple sequence alignment of pheasant *SPRN* gene and Sho protein sequences with those of prion disease-susceptible and -resistant species. In addition, we performed amplicon sequencing of *SPRN* to identify genetic polymorphisms and investigated the genotype, allele and haplotype frequencies as well as linkage disequilibrium (LD) of SNPs of pheasant *SPRN* gene. Furthermore, we evaluated the change of Sho by non-synonymous SNPs in the pheasant *SPRN* gene using *in silico* tools. Lastly, we predicted the secondary and 3D structure of Sho in several prion disease-susceptible and -resistant species using AlphaFold2.

## Materials and methods

### Ethical statements

Tissues of the cerebral cortex from pheasants (*Phasianus colchicus*) (*n* = 135) were collected from slaughterhouses in Korea and stored in a deep freezer (−80°C). The Institutional Animal Care and Use Committee (IACUC) at Jeonbuk National University (JBNU 2020-209) approved this study.

### Genomic DNA

Genomic DNA was extracted from 20 mg cerebral cortex of 135 pheasants by a Bead Genomic DNA Prep kit (Biofact, Daejeon, Korea) according to the manufacturer’s instructions.

### Genetic analysis of the pheasant *SPRN* gene

**Figure 1:**
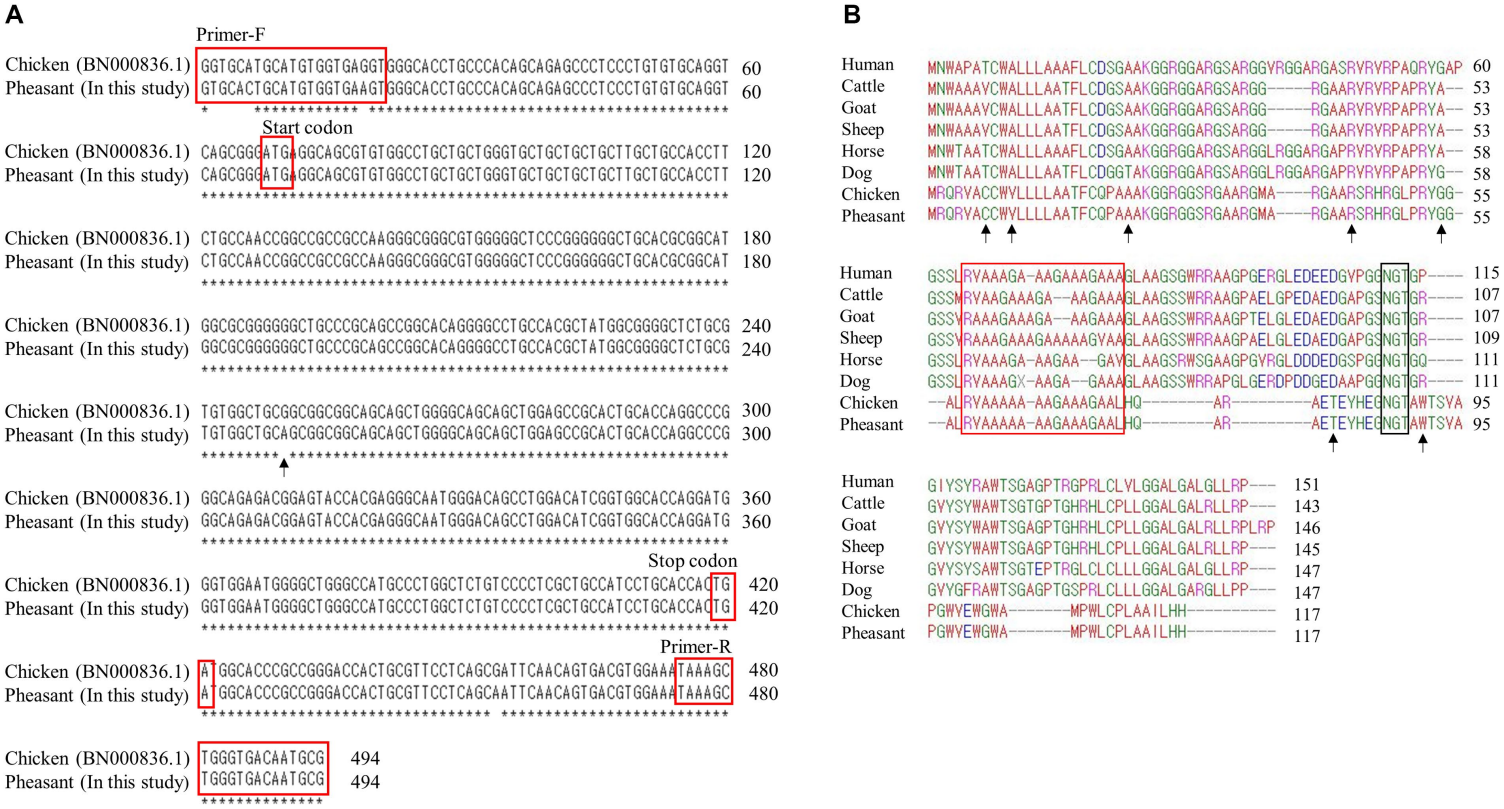
Identification of shadow of prion protein gene (*SPRN*) sequences in pheasants. **(A)** *SPRN* DNA sequence comparison between chicken and pheasant. The arrow indicates the one nucleotide (c.183) difference between chicken and pheasant. **(B)** Multiple sequence alignments of amino acid sequences of the shadow of prion protein (Sho) in various species including humans, cattle, goats, sheep, horses, dogs, chickens, and pheasants. Amino acid chemical properties are indicated by different colors; blue: acidic; red: small and hydrophobic; magenta: basic; green: hydroxyl, sulfhydryl, amine and glycine. The arrows denote the non-synonymous single nucleotide polymorphisms (SNPs) found in this study. The red box highlights the interaction region of Sho with prion protein (PrP) and the black box represents the NXT glycosylation motif of Sho.

To amplify the pheasant *SPRN* gene, a PCR was performed with primers, designed based on the chicken *SPRN* gene (Gene ID: BN000836.1), namely *SPRN*-F (GTGCACTGCATGTGGTGAAGT) and *SPRN*-R (CGCATTGTCACCCAGCTTTA). Detailed information regarding the primers is described in Figure 1A. The 25 μL PCR mixture composed of 2.5 μL of 10× H-star *Taq* reaction buffer, 2.5 μL of 5× band helper, 1 μL of each 10 mM dNTP mix, 1 μL of each primer (10 μM), and 0.2 μL of H-star *Taq* DNA polymerase (BIOFACT, Daejeon, Korea). The following experimental conditions were used for PCR: 98°C for 15 min for denaturation; 40 cycles of 98°C for 20 s, 58°C for 40 s and 72°C for 1 min for denaturation, annealing and extension, respectively and 1 cycle of 72°C for 5 min for the final extension. The PCR products were purified using a FavorPrep GEL/PCR Purification Mini Kit (FAVORGEN, Pingtung City, Taiwan), and sequenced using an ABI 3730xl sequencer (ABI, Foster City, CA, USA). Genotyping was carried out using Finch TV software (Geospiza Inc., Seattle,WA, USA).

To compare linkage between the SNPs of the pheasant’s *PRNP* and *SPRN* genes, data were obtained from our previous study (28). In brief, PCR was performed to amplify the pheasant *PRNP* gene with gene-specific primers, including *PRNP*-F (ATAAAGGAGGTGGGGAT GGG) and *PRNP*-R (CGTGGACACGATGTCATCTC). These primers were designed based on the pheasant *PRNP* gene (Gene ID: 116238382).

### Sequence prediction and multiple sequence alignments analysis

The amplicons of the *SPRN* gene in pheasants were analyzed by a web-based translation tool.^1^ Clustal Omega was used to align the amino acid sequences of Sho. The amino acid sequences of Sho from various species, humans (*Homo sapiens*, NP_001012526.2), cattle (*Bos taurus*, AAY83885.1), goats (*Capra hircus*, AGU17009.1), sheep (*Ovis aries*, NP_001156033.1), horses (*Equus caballus*, XP_023492126.1), dogs (*Canis lupus familiaris*, NC_051832), and chickens (*Gallus gallus*, CAJ43796.1), were obtained from the GenBank of the National Center for Biotechnology Information.

### *In silico* evaluation of the effect of amino acid substitution

The impacts of each amino acid substitution were estimated using the Mutpred2,^2^ MUpro,^3^ and AMYCO *in silico* programs.^4^ MutPred2 is a web server designed to classify mutations as either disease-associated or neutral utilizing a machine-learning-based technique to estimate the molecular mechanism of the pathogenicity of an amino acid substitution. For a pathogenic mutation, the MutPred2 score is higher than 0.5 (34). MUpro can predict alterations in protein stability resulting from missense SNPs. If this tool score predicts a negative delta G (ΔG), it implies that the analyzed SNP may destabilize the protein (35). AMYCO program evaluates the impact of mutation on the aggregation propensity of prion-like domain (PrLD) in prion-like proteins (36).

### 3D structure analysis

The 3D structure of Pheasant *SPRN* was predicted by AlphaFold2, a tool reliant on machine learning.^5^ The confidence of the predicted structure was evaluated by the predicted local distance difference test (pLDDT) value, scaled from 0 to 100. The outcomes of the hydrogen bond alterations from the amino acid substitutions were predicted by the Swiss-PdbViewer (37).^6^

### Statistical analyses

The LD and haplotype analyses were performed using Haploview version 4.2 (Broad Institute, Cambridge, MA, USA). The Hardy–Weinberg Equilibrium (HWE) test was calculated by the chi-square test.

## Results

### First identification of the *SPRN* gene sequence in pheasants

We first performed PCR to amplify the open reading frame (ORF) region of the pheasant *SPRN* gene. We designed *SPRN* gene-specific primers based on the *SPRN* gene sequences of the chicken (*Gallus gallus*) (Figure 1A). The PCR result revealed only one nucleotide difference (c.183) in the ORF of *SPRN* gene between the pheasant and chicken sequences.

### Multiple sequence alignments of Sho among various species

Multiple sequence alignments of amino acid sequences of the Sho protein were performed among humans, cattle, goats, sheep, horses, dogs, chickens, and pheasants (Figure 1B). Pheasants and chickens share the same protein, despite differing by a single nucleotide (c.183). The Sho proteins of pheasants and chickens were the shortest among the eight species (117 amino acids). As in a previous study, although the Sho-PrP interaction regions (red box) and the NXT glycosylation motif (black box) were conserved among every species (14), the N-terminal and C-terminal regions exhibited significantly low sequence homology between mammals and pheasants.

### Identification of novel polymorphisms of the pheasant *SPRN* gene

**Figure 2:**
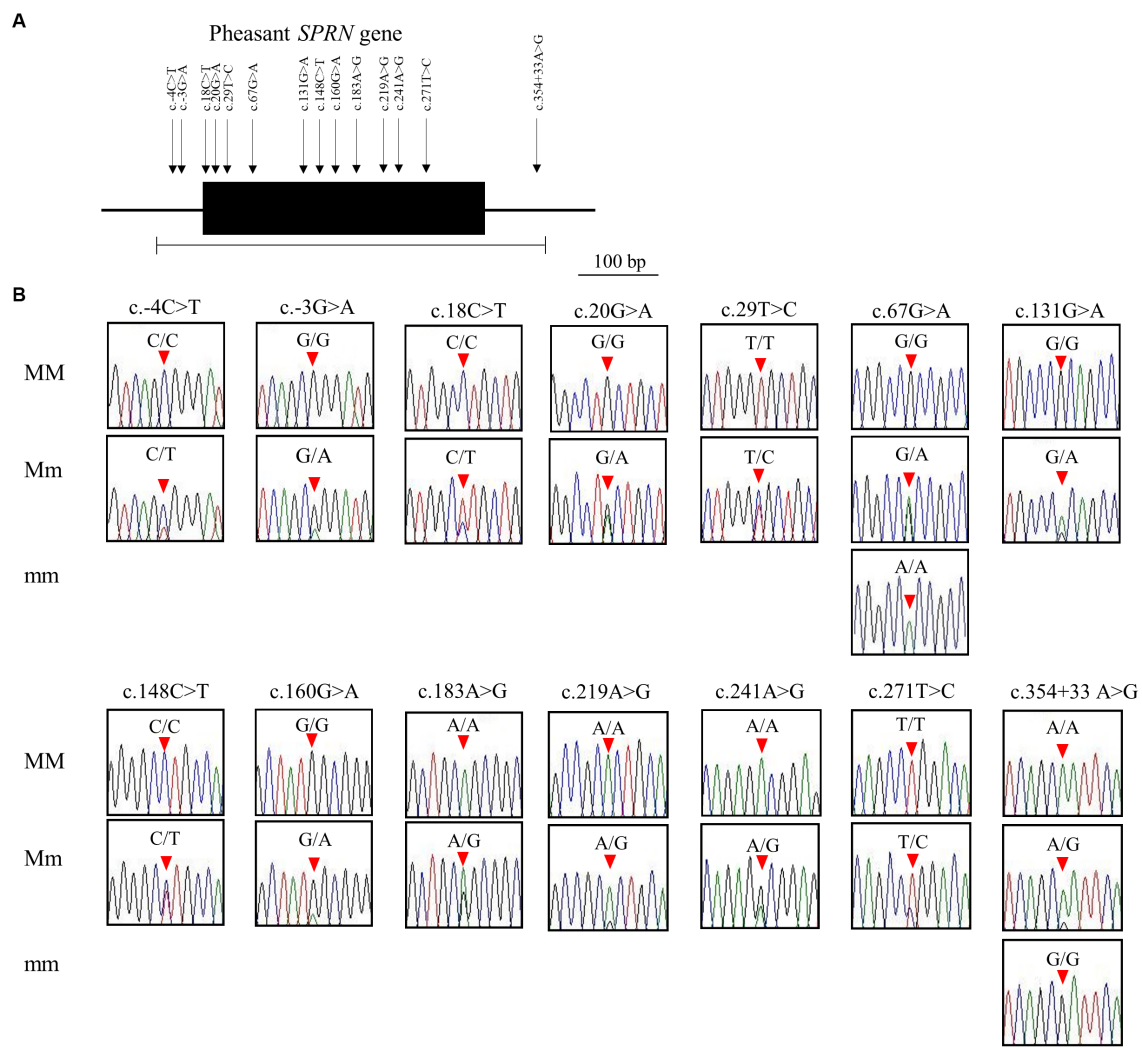
Identification of single nucleotide polymorphisms (SNPs) in pheasant shadow of prion protein gene (*SPRN*). **(A)** Gene map and polymorphisms identified in the *SPRN* in pheasant. The open reading frame (ORF) within exon is marked by a shaded block. Arrows indicate the 14 polymorphisms found in this study. The edged horizontal bar indicates the region sequenced. **(B)** Electropherograms of the 14 novel SNPs of the *SPRN* found in 135 pheasants. The peak colors indicate each base of the DNA sequence (green: adenine; red: thymine; blue: cytosine; black: guanine). The locations of the SNPs found in the present study are indicated by red arrowheads. MM indicates major homozygotes, Mm indicates heterozygotes, and mm indicates minor homozygotes.

We identified a total of 14 novel SNPs: 11 SNPs (c.18C > T (A6A), c.20G > A (C7Y), c.29 T > C (V10A), c.67G > A (A23T), c.131G > A (R44H), c.148C > T (L50L), c.160G > A (G54S), c.183A > G (A61A), c.219A > G (A73A), c.241A > G (T81A), c.271 T > C (W91R)) in the ORF region; 2 SNPs (c.-4C > T, c.-3G > A) upstream of the ORF of the *SPRN* gene; and 1 SNP (c.354 + 33A > G) downstream of the ORF of the *SPRN* gene (Figures 2A,B; Table 1). We found 7 non-synonymous and 4 synonymous SNPs. Detailed values of the genotype and allele frequencies of the SNPs in the pheasant *SPRN* gene are described in Table 1.

**Table 1:** Genotype and allele frequencies of shadow of prion protein gene (*SPRN*) polymorphisms in 135 pheasants.

Polymorphisms	Genotype frequencies, *n* (%)			Allele frequencies, *n* (%)		HWE
	MM	Mm	mm	M	m	
c.-4C > T	134 (99.3)	1 (0.7)	0 (0)	269 (99.6)	1 (0.4)	0.9655
c.-3G > A	134 (99.3)	1 (0.7)	0 (0)	269 (99.6)	1 (0.4)	0.9655
c.18C > T	133 (98.5)	2 (1.5)	0 (0)	268 (99.3)	2 (0.7)	0.9309
c.20G > A (C7Y)	134 (99.3)	1 (0.7)	0 (0)	269 (99.6)	1 (0.4)	0.9655
c.29 T > C (V10A)	133 (98.5)	2 (1.5)	0(0)	268 (99.3)	2 (0.7)	0.9309
c.67G > A (A23T)	133 (98.5)	1 (0.7)	1 (0.7)	267 (98.9)	3 (1.1)	0.000
c.131G > A (R44H)	134 (99.3)	1 (0.7)	0 (0)	269 (99.6)	1 (0.4)	0.9655
c.148C > T	134 (99.3)	1 (0.7)	0 (0)	269 (99.6)	1 (0.4)	0.9655
c.160G > A (G54S)	134 (99.3)	1 (0.7)	0 (0)	269 (99.6)	1 (0.4)	0.9655
c.183A > G	129 (95.6)	6 (4.4)	0 (0)	264 (97.8)	6 (2.2)	0.7917
c.219A > G	133 (98.5)	2(1.5)	0(0)	268(99.3)	2(0.7)	0.9309
c.241A > G (T81A)	133 (98.5)	2 (1.5)	0 (0)	268 (99.3)	2 (0.7)	0.9309
c.271 T > C (W91R)	134 (99.3)	1 (0.7)	0 (0)	269 (99.6)	1 (0.4)	0.9655
c.354 + 33A > G	128 (94.8)	6 (4.4)	1 (0.7)	262 (97.0)	8 (3.0)	0.0083

MM, Major homozygote; Mm, Heterozygote; mm, Minor homozygote; HWE, Hardy–Weinberg equilibrium; M, Major allele; m, Minor allele.

We also analyzed the LD among all polymorphisms of the pheasant *SPRN* gene with their *r*^2^ values (Table 2). A total of 5 strong LDs (*r*^2^ > 0.333) were found. In addition, we investigated LD values between *PRNP* and *SPRN* polymorphisms. Strong LD (*r*^2^ > 0.333) was not observed between *PRNP* and *SPRN* polymorphisms in pheasants (Table 3). Furthermore, we conducted a haplotype analysis of all the polymorphisms found in the *SPRN* gene of the pheasant (Table 4). Three major haplotypes were identified in pheasants. The CGCGTGGCGAAATA (haplotype1, HT1) was most frequently observed (93.3%) in the pheasant *SPRN* gene, followed by CGCGTAGCGAAATA (haplotype2, HT2) (1.1%) and CGCGTGGCGAAATG (haplotype3, HT3) (1.1%).

**Table 2:** Linkage disequilibrium (LD) analysis of the 14 *SPRN* polymorphisms of pheasants.

	c.-4C > T	c.-3G > A	c.18C > T	c.20G > A	c.29 T > C	c.67G > A	c.131G > A	c.148C > T	c.160G > A	c.183A > G	c.219A > G	c.241A > G	c.271 T > C	354 + 33A > G
c.-4C > T	–													
c.-3G > A	0.0	–												
c.18C > T	**0.498**	0.0	–											
c.20G > A	0.0	0.0	0.0	–										
c.29 T > C	0.0	0.0	0.0	0.0	–									
c.67G > A	0.0	0.0	0.0	0.0	0.0	–								
c.131G > A	0.0	0.0	**0.498**	0.0	0.0	0.0	–							
c.148C > T	0.0	0.0	0.0	0.0	0.0	0.0	0.0	–						
c.160G > A	0.0	0.0	**0.498**	0.0	0.0	0.0	**1**	0.0	–					
c.183A > G	0.164	0.164	0.075	0.0	0.075	0.0	0.0	0.164	0.0	–				
c.219A > G	0.0	0.0	0.0	0.0	0.0	0.0	0.0	0.0	0.0	0.075	–			
c.241A > G	0.0	0.0	0.0	0.0	**1**	0.0	0.0	0.0	0.0	0.075	0.0	–		
c.271 T > C	0.0	0.0	0.0	0.0	0.0	0.0	0.0	0.0	0.0	0.0	0.0	0.0	–	
c.354 + 33A > G	0.122	0.122	0.054	0.0	0.057	0.0	0.0	0.0	0.0	0.317	0.0	0.057	0.0	–

Bold text indicates strong LD (*r*^2^ > 0.333).

**Table 3:** Linkage disequilibrium (LD) analysis between polymorphisms in the *PRNP* and *SPRN* genes in pheasants.

	c.-4C > T	c.-3G > A	c.18C > T	c.20G > A	c.29 T > C	c.67G > A	c.131G > A	c.148C > T	c.160G > A	c.183A > G	c.219A > G	c.241A > G	c.271 T > C	354 + 33A > G
c.61G > T	0.0	0.0	0.0	0.0	0.075	0.0	0.0	0.0	0.0	0.017	0.0	0.075	0.0	0.015
c.67C > T	0.0	0.0	0.0	0.0	0.0	0.0	0.0	0.0	0.0	0.001	0.0	0.0	0.0	0.001
c.97G > A	0.0	0.0	0.0	0.0	0.0	0.0	0.0	0.0	0.0	0.0	0.0	0.0	0.0	0.0
Ins/del type 1	0.0	0.0	0.0	0.0	0.0	0.0	0.0	0.0	0.0	0.0	0.0	0.0	0.0	0.0
Ins/del type 2	0.001	0.001	0.0	0.001	0.003	0.0	0.010	0.010	0.010	0.001	0.0	0.003	0.001	0.012
Ins/del type 3	0.0	0.0	0.0	0.0	0.0	0.0	0.0	0.0	0.0	0.0	0.0	0.0	0.0	0.0
Ins/del type 4	0.0	0.0	0.011	0.0	0.011	0.007	0.036	0.0	0.036	0.007	0.011	0.011	0.0	0.005
Ins/del type 5	0.0	0.0	0.011	0.0	0.011	0.007	0.036	0.0	0.036	0.007	0.011	0.011	0.0	0.005
c.530G > A	0.042	0.0	0.013	0.0	0.013	0.001	0.0	0.0	0.0	0.010	0.001	0.013	0.042	0.003
Ins/del type 6	0.0	0.0	0.0	0.0	0.0	0.0	0.0	0.0	0.0	0.0	0.0	0.0	0.0	0.001
c.750C > G	0.002	0.002	0.003	0.009	0.003	0.0	0.002	0.002	0.002	0.01	0.003	0.003	0.009	0.005
c.766G > A	0.001	0.001	0.002	0.001	0.002	0.003	0.001	0.001	0.001	0.005	0.002	0.002	0.017	0.007
c.781G > A	0.002	0.002	0.001	0.002	0.001	0.008	0.009	0.009	0.009	0.003	0.019	0.001	0.002	0.003

The vertical and horizontal axes represent *PRNP* and *SPRN* polymorphisms, respectively. The LD analysis was performed using the non-synonymous SNPs of pheasant *PRNP* gene. Ins/del Type 1: c.163_180delAACCCGGGGTATCCCCAC, Ins/del Type 2: c.180_181insAACCCGGGGTATCCCCAC, Ins/del Type 3: c.180_181insAACCCGGGGTATCCCCACAACCCGGGGTATCCCCAC, Ins/del Type 4: c.198_199insAACCCAGGATATCCCCAC, Ins/del Type 5: c.216_217insAACCCGGCTATCCCCACAACCCCGGCTATCCCCAC, Ins/del Type 6: c.624_626delGAA.

**Table 4:** Haplotype frequency of 14 *SPRN* polymorphisms in pheasants.

Haplotype	Frequency, *n* (%)
HT1 CGCGTGGCGAAATA	252 (0.933)
HT2 CGCGTAGCGAAATA	3 (0.011)
HT3 CGCGTGGCGAAATG	3 (0.011)
Others	12 (0.045)
Total	270 (1.0)

HT: haplotype. Others contains rare haplotypes with frequency < 0.01.

### The comparison of number of SNPs of the SPRN gene in prion disease-susceptible and prion disease-resistant species

**Figure 3:**
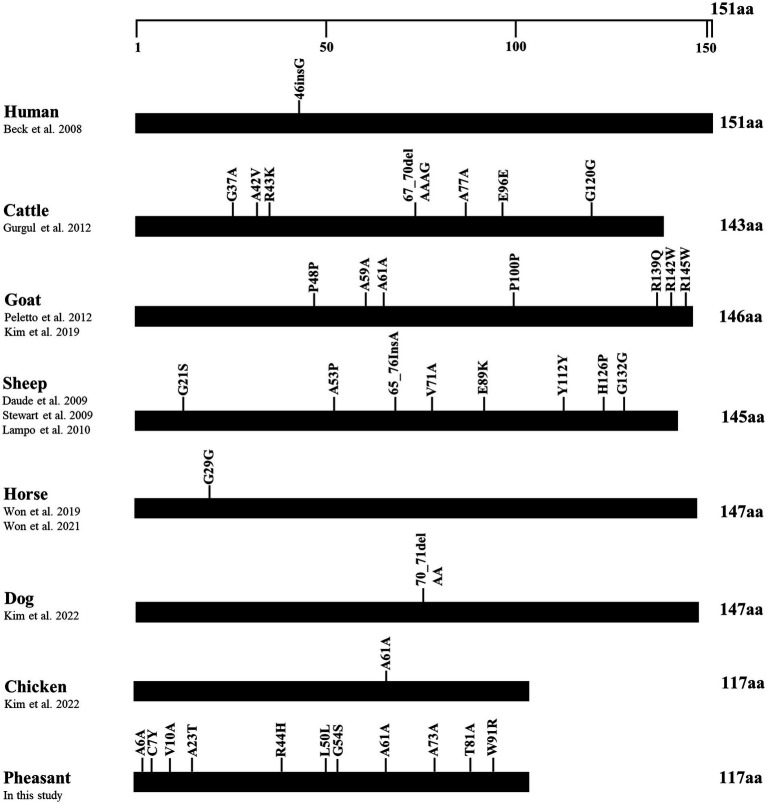
Distribution of genetic polymorphisms in the open reading frame (ORF) of the shadow of prion protein gene (*SPRN*) in various species. The figure shows previously reported genetic polymorphisms of the *SPRN* gene in a multitude of species such as humans, cattle, goats, sheep, horses, dogs, and chickens, as well as pheasants reported in this study. The edged horizontal bar signifies the length of the amino acids in the *SPRN*.

To identify differences in the number of SNPs between pheasants and other species, we collected polymorphisms in the ORF of the *SPRN* gene from prion disease-susceptible (human, cattle, goat, sheep) and prion disease-resistant (horse, dog, chicken) species. Notably, the prion disease-susceptible species exhibited several genetic polymorphisms that lead to amino acid changes in the ORF of the *SPRN* gene. Three non-synonymous SNPs were reported in cattle and goats, and five non-synonymous SNPs were reported in sheep. However, only one synonymous SNP was identified in prion-resistant species, such as horses and chickens. In this study, we identified several genetic polymorphisms, including 7 non-synonymous and 4 synonymous SNPs, in pheasants (Figure 3).

### *In silico* analysis of the effect of polymorphisms in the pheasant *SPRN* gene

To analyze the functional and structural effects of non-synonymous SNPs in the pheasant Sho, we used MutPred2 and MUpro (Table 5). According to the MutPred2 analysis, the 7 non-synonymous SNPs had scores less than 0.5, indicating benign effects. However, the MUpro analysis gave these 7 non-synonymous SNPs scores below 0.0, indicating a decrease in protein stability (Table 5). We used AMYCO to analyze the effects of the 7 non-synonymous SNPs on the amyloid propensity of pheasant Sho. The pheasant Sho sequences containing the 7 non-synonymous SNPs had a score of 0.0, which is identical to that of the wild-type pheasant Sho (Table 5).

**Table 5:** *In silico* evaluation on effect of non-synonymous single nucleotide polymorphisms (SNPs) in the pheasant.

Polymorphisms	MutPred2	MUpro		AMYCO
		Score	Stability	
c.20G > A (C7Y)	0.041	−1.06562	Decrease	0.0
c.29 T > C (V10A)	0.022	−1.22098	Decrease	0.0
c.67G > A (A23T)	0.058	−1.18032	Decrease	0.0
c.131G > A (R44H)	0.023	−1.01590	Decrease	0.0
c.160G > A (G54S)	0.039	−0.36096	Decrease	0.0
c.241A > G (T81A)	0.029	−0.82047	Decrease	0.0
c.271 T > C (W91R)	0.093	−1.11327	Decrease	0.0

### Prediction of the 3D structure of Sho among various species using AlphaFold2

**Figure 4:**
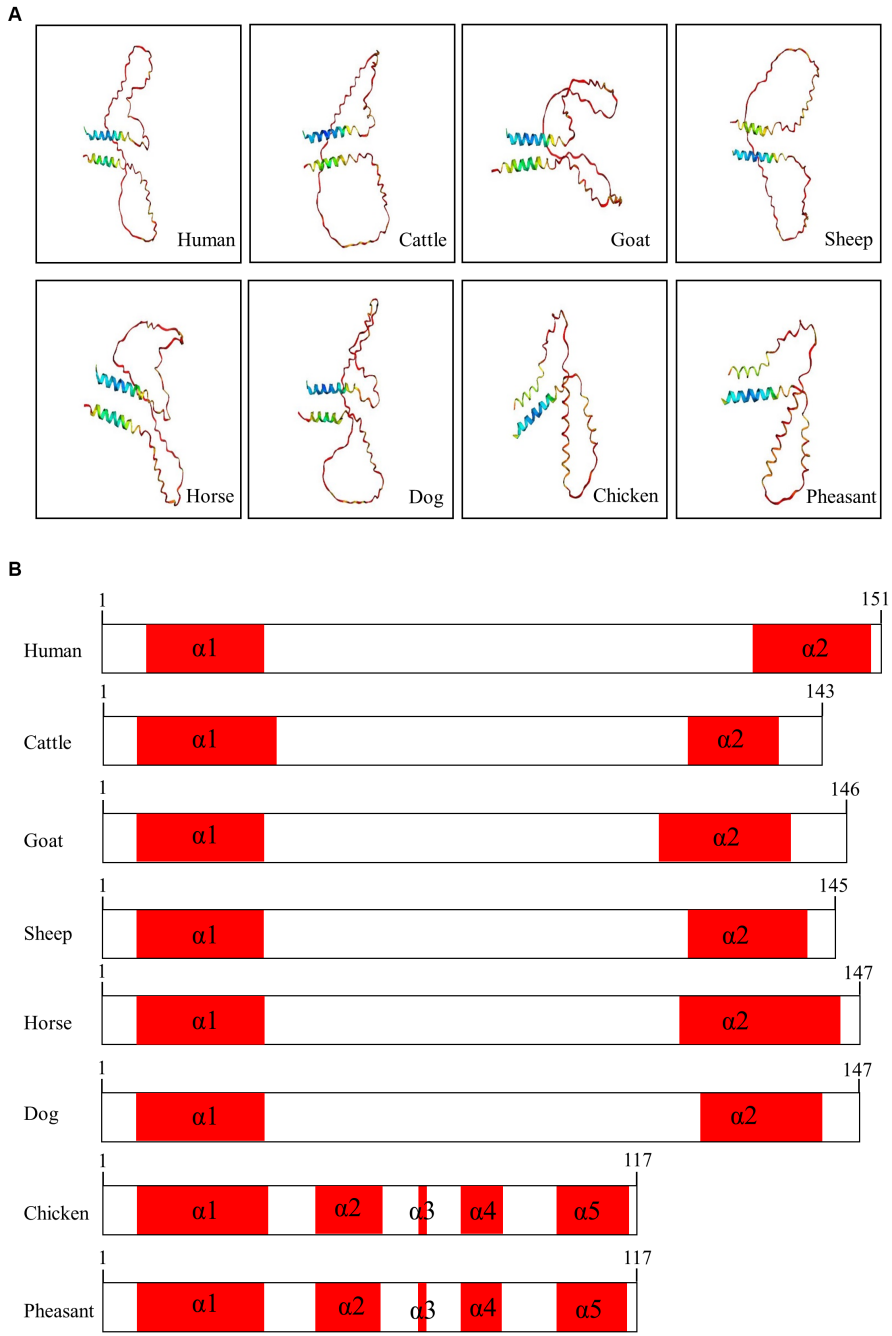
Prediction of 3D and secondary structures of the shadow of prion protein (Sho) in various species. **(A)** The 3D structures of human, cattle, goat, sheep, horse, dog, chicken, and pheasant Sho were analyzed by Alphafold2. **(B)** The secondary structures of human, cattle, goat, sheep, horse, dog, chicken, and pheasant Sho. In the structures, α-helices are represented in red, and coils are represented in white.

**Figure 5:**
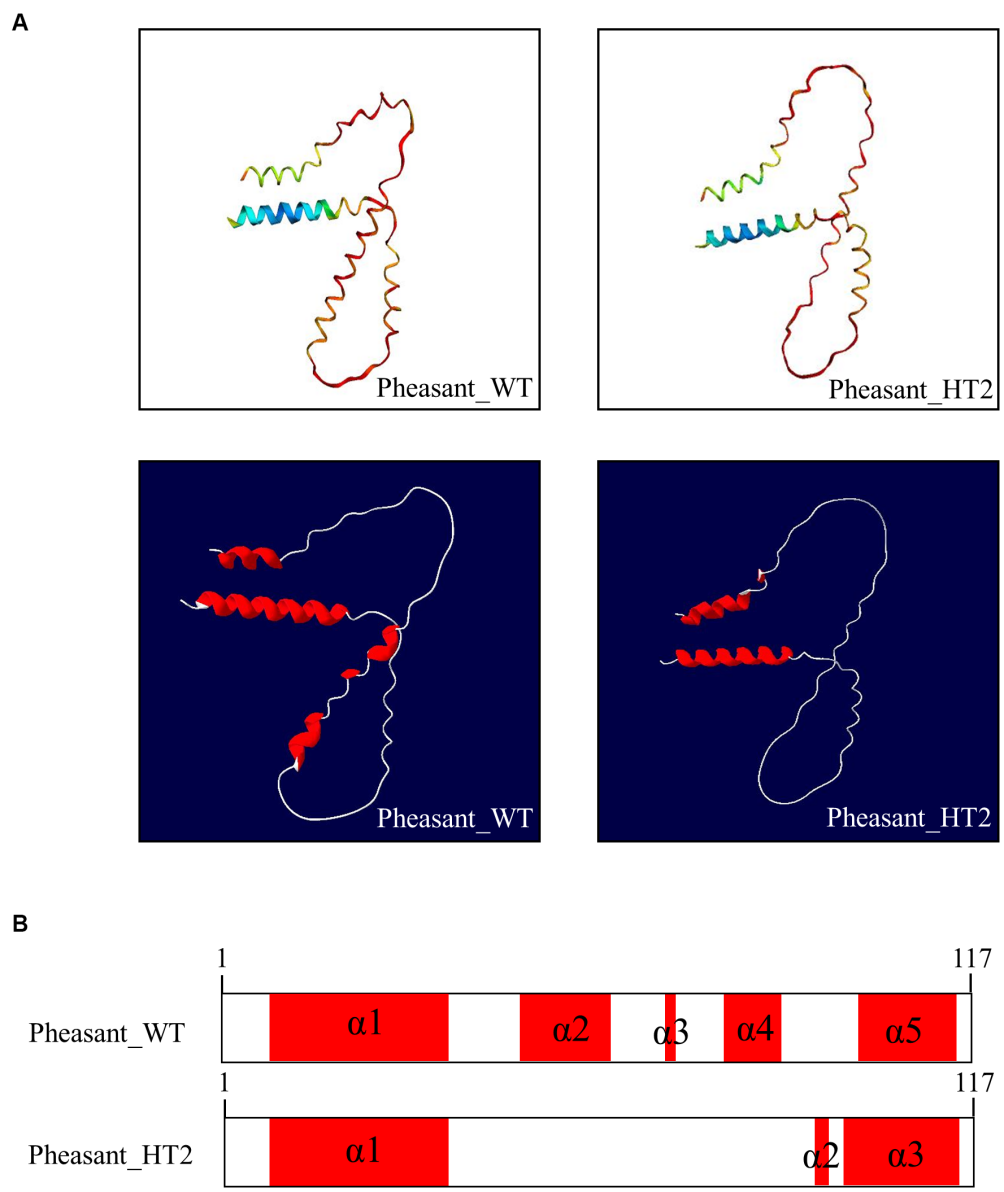
Prediction of 3D and secondary structures of the shadow of prion protein (Sho) in pheasants. **(A)** The 3D structures of wild-type and CGCGTAGCGAAATA (haplotype2, HT2) pheasant Sho. **(B)** The secondary structures of wild-type and CGCGTAGCGAAATA (haplotype2, HT2) pheasant Sho. In the structures, α-helices are represented in red, and coils are represented in white.

We used AlphaFold2 to analyze the 3D structures of Sho in eight species: humans, cattle, goats, sheep, horses, dogs, chickens, and pheasants. The structures of chicken and pheasant exhibited the same shape (Figure 4A). Two α-helices were predicted to be linked with the coil in Sho from all species except chicken and pheasant Sho. However, chicken and pheasant Sho proteins were predicted to have five α-helices (codons 3–22, 57–63, 70, 74–77 and 107–115) (Figure 4B). In addition, we predicted the 3D structure of the pheasant Sho carrying the CGCGTAGCGAAATA (haplotype2, HT2) and found it to be distinct from the wild type (Figure 5A). Pheasant Sho carrying the HT2 has three α-helices (codons 3–22, 102 and 106–115) (Figure 5B). Pheasant Sho carrying HT1 is the same as the wild-type, and HT3 is in the downstream of the ORF of the *SPRN* gene. HT3 has no effect on the structure of the Sho protein.

### Prediction of the structural alteration of pheasant Sho induced by non-synonymous SNPs

**Figure 6:**
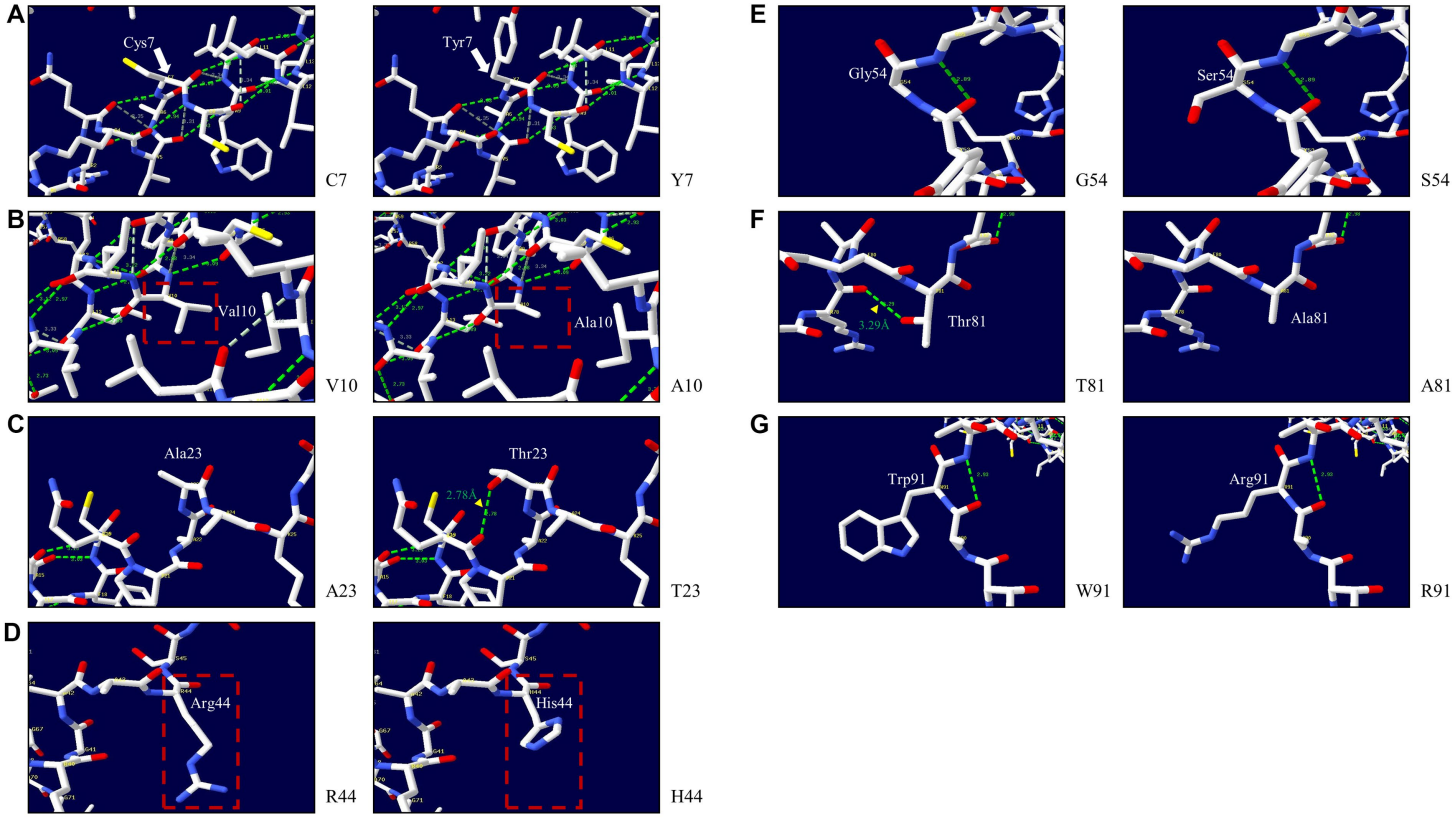
Prediction of the 3D structure and hydrogen bonds of the shadow of prion protein (Sho) in pheasants. **(A)** 3D structure of pheasant Sho carrying the C7 and Y7 alleles. **(B)** 3D structure of pheasant Sho carrying the V10 and A10 alleles. **(C)** 3D structure of pheasant Sho carrying the A23 and T23 alleles. **(D)** 3D structure of pheasant Sho carrying the R44 and H44 alleles. **(E)** 3D structure of pheasant Sho carrying the G54 and S54 alleles. **(F)** 3D structure of pheasant Sho carrying the T81 and A81 alleles. **(G)** 3D structure of pheasant Sho carrying the W91 and R91 alleles.

We explored the effects of 7 non-synonymous SNPs on the 3D structure of the pheasant Sho protein (Figure 6). First, the 3D structures of wild-type pheasant Sho were predicted by AlphaFold2. Then, the predicted structure was pictured using Swiss-PdbViewer, and the impact of 7 non-synonymous SNPs on pheasant Sho protein was analyzed. The C7 and Y7 alleles have three hydrogen bonds of the same length (Figure 6A). The V10 and A10 alleles have three hydrogen bonds of the same length (Figure 6B). Hydrogen bonds were absent in the A23 allele, but the T23 allele showed a hydrogen bond with Q20 (2.78 Å) (Figure 6C). Hydrogen bonds were absent in the R44, H44, G54 and S54 alleles (Figures 6D,E). The T81 allele showed a hydrogen bond with R78 (3.29 Å), but a hydrogen bond was absent in the A81 allele (Figure 6F). The W91 and R91 alleles also did not have hydrogen bonds (Figure 6G).

## Discussion

Considering that chickens are known to be resistant to prion diseases, it becomes crucial to investigate the genes associated with prion disease in pheasants. In the present study, we are the first to report pheasant *SPRN* gene sequences (Figure 1A). Chicken and pheasant have one nucleotide difference, but they share the same amino acid sequence. To identify the specific features of the pheasant Sho protein, we conducted a comparison of the amino acid sequences of the Sho from a variety of species and found that major components of Sho protein including prion interaction region and NXT glycosylation motif were conserved in pheasant like other prion-related species (Figure 1B).

In addition, we found 14 novel SNPs, including 7 non-synonymous and 4 synonymous SNPs, using amplicon sequencing (Figure 2). Previous studies have reported that prion disease-susceptible species, including cattle, goats, and sheep, have highly polymorphic *SPRN* genes (12, 13, 38, 39). In contrast, a prion disease-resistant species, horses and chickens have one polymorphism of the *SPRN* gene (9, 14, 40). Dogs, a prion disease-resistant animal, have one insertion/deletion polymorphism in *SPRN* gene (10). Interestingly, insertion/deletion polymorphisms have not been identified in the ORF of the pheasant *SPRN* gene. Although, phylogenetic analysis revealed that pheasants are closely related to chickens, a prion-resistant species (22–24), our results showed that pheasants have many polymorphisms. Further research is necessary to explore the *SPRN* gene in other species and to investigate the relationship between the number of *SPRN* gene polymorphisms and susceptibility to prion diseases. This is important because pathogenic mutations have a higher likelihood of occurring in *SPRN* genes that exhibit high polymorphism, as reported previously (10). Moreover, *SPRN* SNPs with low frequency have been discovered. Although the pheasant Sho sequence was identical to chicken Sho, the presence of a significant number of SNPs, albeit at a low frequency, can lead to structural abnormalities in the prion protein that encodes the prion-related protein. Therefore, further study is needed to comprehend how these characteristics could contribute to the progression of prion diseases and determine their impact on the prion pathomechanism.

To evaluate the impact of amino acid substitutions, we employed *in silico* programs (Table 5). The MutPred2 scores for all 7 non-synonymous SNPs were below 0.5, indicating no pathogenicity. We used MUpro to predict the effects of the SNPs on protein stability and found that a decrease in protein stability was associated with the 7 non-synonymous SNPs. Prion diseases are characterized by protein misfolding, and previous research has identified two important residues in PrP that promote stability in dogs and horses, animals known to be resistant to prion diseases (41). Those residues induce local changes that decrease the β-sheet content and enhance the structural stability of horse and dog PrP^C^, allowing the changes to spread to nearby regions (41). Further research is needed to understand how the decreased stability of the protein, in addition to its association with accelerated prion formation, can be interpreted in relation to the intrinsic functions of the Sho protein, such as embryo development. In addition, we employed AMYCO to estimate the impact of the mutations on the aggregation propensity of the PrLDs in prion-like proteins. Protein aggregation is a characteristic observed in various neurodegenerative disorders (42). The pheasant Sho sequences, when substituted with 7 non-synonymous SNPs, exhibited a score of 0.0, suggesting that the 7 non-synonymous SNPs do not have any amyloid-prone features.

We analyzed the 3D structures of Sho among various species predicted by AlphaFold2. Interestingly, with the exception of chicken and pheasant Sho, all species displayed a similar 3D structure, and none of the Sho proteins from any species exhibited a β-sheet (Figure 4). Additionally, we analyzed pheasant Sho according to the haplotype of pheasant Sho (Figure 5). By comparing the 3D structures of the pheasant Sho with both the wild-type allele and an HT2 variant, we observed a decrease in the number of α-helices in the pheasant Sho with HT2. The presence of HT1 and HT3 did not seem to impact the structure of the Sho protein. Since Sho plays a vital role in embryonic development, gaining insights into its structure can greatly contribute to comprehending its functional mechanisms (17, 43). Research is needed to investigate how structural changes in the haplotype affect the general function of Sho and its ability to interact with PrP^C^.

## Conclusion

In this study, we first reported pheasant *SPRN* gene sequences and found 14 novel SNPs of the pheasant *SPRN* gene, including 7 non-synonymous and 4 synonymous in a total of 135 pheasants. *In silico* analysis, it was predicted that 7 non-synonymous SNPs induce decrease of protein stability. In addition, the secondary and tertiary structures of the wild-type pheasant Sho are identical to those of the chicken Sho. To the best of our knowledge, this is the first study on genetic polymorphisms of the pheasant *SPRN* gene.

## Data availability statement

The data presented in the study are deposited in the DRYAD repository https://datadryad.org/stash/share/9SErCsr_yxn1mmXQBsndJYntnvhR-sCJo-sjjnyumeE.

## Ethics statement

The animal study was approved by the Institutional Animal Care and Use Committee (IACUC) at Jeonbuk National University (JBNU 2020-209). The study was conducted in accordance with the local legislation and institutional requirements.

## Author contributions

D-IC: Conceptualization, Visualization, Writing – original draft. MZ: Formal analysis, Writing – review & editing. Y-CK: Formal analysis, Writing – review & editing. B-HJ: Conceptualization, Formal analysis, Writing – review & editing.

## Footnotes

^1^ https://web.expasy.org/translate/

^2^ http://mutpred.mutdb.org/

^3^ http://mupro.proteomics.ics.uci.edu/

^4^ http://bioinf.uab.cat/amyco/

^5^ https://colab.research.google.com/github/sokrypton/ColabFold/blob/v1.2.0/AlphaFold2.ipynb

^6^ https://spdbv.unil.ch/

## References

[1] Prusiner SB. Prions. Proc Natl Acad Sci USA. (1998) 95:13363–83. doi: 10.1073/pnas.95.23.13363, PMID: 9811807 PMC33918

[2] Kim YC Lee J Lee DW Jeong BH. Large-scale lipidomic profiling identifies novel potential biomarkers for prion diseases and highlights lipid raft-related pathways. Vet Res. (2021) 52:105. doi: 10.1186/s13567-021-00975-1, PMID: 34289911 PMC8296529

[3] Fernández-Borges N Parra B Vidal E Eraña H Sánchez-Martín MA de Castro J. Unraveling the key to the resistance of canids to prion diseases. PLoS Pathog. (2017) 13:e1006716. doi: 10.1371/journal.ppat.1006716, PMID: 29131852 PMC5703577

[4] Wells GA Scott AC Johnson CT Gunning RF Hancock RD Jeffrey M. A novel progressive spongiform encephalopathy in cattle. Vet Rec. (1987) 121:419–20. doi: 10.1136/vr.121.18.419, PMID: 3424605

[5] Bons N Mestre-Frances N Belli P Cathala F Gajdusek DC Brown P. Natural and experimental oral infection of nonhuman primates by bovine spongiform encephalopathy agents. Proc Natl Acad Sci USA. (1999) 96:4046–51. doi: 10.1073/pnas.96.7.4046, PMID: 10097160 PMC22417

[6] Zeineldin M Cox-Struble H Camp P Farrell D Pritchard R Thacker TC. National Prevalence of caprine prion protein genetic variability at codons 146, 211, and 222 in goat herds in the United States. Vet Sci. (2023) 11:13. doi: 10.3390/vetsci11010013, PMID: 38250919 PMC10818752

[7] Benestad SL Telling GC. Chronic wasting disease: an evolving prion disease of cervids. Handb Clin Neurol. (2018) 153:135–51. doi: 10.1016/b978-0-444-63945-5.00008-8, PMID: 29887133

[8] Iulini B Cantile C Mandara MT Maurella C Loria GR Castagnaro M. Neuropathology of italian cats in feline spongiform encephalopathy surveillance. Vet Pathol. (2008) 45:626–33. doi: 10.1354/vp.45-5-626, PMID: 18725465

[9] Won SY Kim YC Do K Jeong BH. The first report of genetic polymorphisms of the equine SPRN gene in outbred horses, Jeju and Halla horses. Animals. (2021) 11:2574. doi: 10.3390/ani11092574, PMID: 34573540 PMC8467739

[10] Kim YC Kim HH Kim AD Jeong BH. Novel insertion/deletion polymorphisms and genetic features of the shadow of prion protein gene (SPRN) in dogs, a prion-resistant animal. Front Vet Sci. (2022) 9:942289. doi: 10.3389/fvets.2022.942289, PMID: 35982928 PMC9378991

[11] Moore J Hawkins SA Austin AR Konold T Green RB Blamire IW. Studies of the transmissibility of the agent of bovine spongiform encephalopathy to the domestic chicken. BMC Res Notes. (2011) 4:501. doi: 10.1186/1756-0500-4-501, PMID: 22093239 PMC3341577

[12] Kim YC Kim SK Won SY Jeong BH. Polymorphisms of shadow of prion protein gene (SPRN) in Korean native cattle (Hanwoo) and Holstein cattle. Sci Rep. (2020) 10:15272. doi: 10.1038/s41598-020-72225-x, PMID: 32943703 PMC7499179

[13] Kim YC Kim SK Jeong BH. Scrapie susceptibility-associated indel polymorphism of shadow of prion protein gene (SPRN) in Korean native black goats. Sci Rep. (2019) 9:15261. doi: 10.1038/s41598-019-51625-8, PMID: 31649311 PMC6813300

[14] Kim YC Kim HH Jeong BH. The first report of polymorphisms and genetic characteristics of the shadow of prion protein (SPRN) in prion disease-resistant animal, chickens. Front Vet Sci. (2022) 9:904305. doi: 10.3389/fvets.2022.904305, PMID: 35782543 PMC9247643

[15] Onodera T Nishimura T Sugiura K Sakudo A. Function of prion protein and the family member, Shadoo. Curr Issues Mol Biol. (2020) 36:67–88. doi: 10.21775/cimb.036.067 31559969

[16] Daude N Westaway D. Biological properties of the PrP-like Shadoo protein. Front Biosci. (2011) 16:1505–16. doi: 10.2741/3801, PMID: 21196244

[17] Passet B Castille J Makhzami S Truchet S Vaiman A Floriot S. The prion-like protein Shadoo is involved in mouse embryonic and mammary development and differentiation. Sci Rep. (2020) 10:6765. doi: 10.1038/s41598-020-63805-y, PMID: 32317725 PMC7174383

[18] Premzl M Sangiorgio L Strumbo B Marshall Graves JA Simonic T Gready JE. Shadoo, a new protein highly conserved from fish to mammals and with similarity to prion protein. Gene. (2003) 314:89–102. doi: 10.1016/s0378-1119(03)00707-8, PMID: 14527721

[19] Ciric D Richard CA Moudjou M Chapuis J Sibille P Daude N. Interaction between Shadoo and PrP affects the PrP-folding pathway. J Virol. (2015) 89:6287–93. doi: 10.1128/jvi.03429-14, PMID: 25855735 PMC4474288

[20] Allais-Bonnet A Pailhoux E. Role of the prion protein family in the gonads. Front Cell Dev Biol. (2014) 2:56. doi: 10.3389/fcell.2014.00056, PMID: 25364761 PMC4207050

[21] Makzhami S Passet B Halliez S Castille J Moazami-Goudarzi K Duchesne A. The prion protein family: a view from the placenta. Front Cell Dev Biol. (2014) 2:35. doi: 10.3389/fcell.2014.00035, PMID: 25364742 PMC4207016

[22] Prum RO Berv JS Dornburg A Field DJ Townsend JP Lemmon EM. A comprehensive phylogeny of birds (Aves) using targeted next-generation DNA sequencing. Nature. (2015) 526:569–73. doi: 10.1038/nature15697, PMID: 26444237

[23] Helm-Bychowski KM Wilson AC. Rates of nuclear DNA evolution in pheasant-like birds: evidence from restriction maps. Proc Natl Acad Sci USA. (1986) 83:688–92. doi: 10.1073/pnas.83.3.688, PMID: 3003745 PMC322929

[24] van Raamsdonk LWD Prins TW Meijer N Scholtens IMJ Bremer M de Jong J. Bridging legal requirements and analytical methods: a review of monitoring opportunities of animal proteins in feed. Food Addit Contam Part A Chem Anal Control Expo Risk Assess. (2019) 36:46–73. doi: 10.1080/19440049.2018.1543956, PMID: 30608892

[25] Ding J Jiang T Zhou H Yang L He C Xu K. The gut microbiota of pheasant lineages reflects their host genetic variation. Front Genet. (2020) 11:859. doi: 10.3389/fgene.2020.00859, PMID: 32903781 PMC7438946

[26] Gibbs CJ Jr Gajdusek DC. Experimental subacute spongiform virus encephalopathies in primates and other laboratory animals. Science. (1973) 182:67–8. doi: 10.1126/science.182.4107.67, PMID: 4199733

[27] Barlow RM Rennie JC. The fate of ME7 scrapie infection in rats, guinea-pigs and rabbits. Res Vet Sci. (1976) 21:110–1. doi: 10.1016/S0034-5288(18)33406-4, PMID: 821118

[28] Kim KH Kim YC Jeong BH. Novel polymorphisms and genetic characteristics of the prion protein gene in pheasants. Front Vet Sci. (2022) 9:935476. doi: 10.3389/fvets.2022.935476, PMID: 35903139 PMC9322948

[29] Chiarella P Capone P Sisto R. Contribution of genetic polymorphisms in human health. Int J Environ Res Public Health. (2023) 20:912. doi: 10.3390/ijerph20020912, PMID: 36673670 PMC9858723

[30] Kim YC Kim HH Kim K Kim AD Jeong BH. Novel polymorphisms and genetic characteristics of the shadow of prion protein gene (SPRN) in cats, hosts of feline spongiform encephalopathy. Viruses. (2022) 14:981. doi: 10.3390/v14050981, PMID: 35632724 PMC9148082

[31] Gurgul A Polak MP Larska M Słota E. PRNP and SPRN genes polymorphism in atypical bovine spongiform encephalopathy cases diagnosed in polish cattle. J Appl Genet. (2012) 53:337–42. doi: 10.1007/s13353-012-0102-4, PMID: 22723200

[32] Beck JA Campbell TA Adamson G Poulter M Uphill JB Molou E. Association of a null allele of SPRN with variant Creutzfeldt-Jakob disease. J Med Genet. (2008) 45:813–7. doi: 10.1136/jmg.2008.061804, PMID: 18805828 PMC2590874

[33] Peletto S Bertolini S Maniaci MG Colussi S Modesto P Biolatti C. Association of an indel polymorphism in the 3'UTR of the caprine SPRN gene with scrapie positivity in the central nervous system. J Gen Virol. (2012) 93:1620–3. doi: 10.1099/vir.0.041400-0, PMID: 22492914

[34] Choudhury A Mohammad T Anjum F Shafie A Singh IK Abdullaev B. Comparative analysis of web-based programs for single amino acid substitutions in proteins. PLoS One. (2022) 17:e0267084. doi: 10.1371/journal.pone.0267084, PMID: 35507592 PMC9067658

[35] Kalmari A Hosseinzadeh Colagar A Heydari M Arash V. Missense polymorphisms potentially involved in mandibular prognathism. J Oral Biol Craniofac Res. (2023) 13:453–60. doi: 10.1016/j.jobcr.2023.05.007, PMID: 37228872 PMC10203774

[36] Iglesias V Conchillo-Sole O Batlle C Ventura S. AMYCO: evaluation of mutational impact on prion-like proteins aggregation propensity. BMC Bioinform. (2019) 20:24. doi: 10.1186/s12859-019-2601-3, PMID: 30642249 PMC6332698

[37] Guex N Peitsch MC. SWISS-MODEL and the Swiss-PdbViewer: an environment for comparative protein modeling. Electrophoresis. (1997) 18:2714–23. doi: 10.1002/elps.1150181505, PMID: 9504803

[38] Lampo E Van Poucke M Hugot K Hayes H Van Zeveren A Peelman LJ. Characterization of the genomic region containing the shadow of prion protein (SPRN) gene in sheep. BMC Genomics. (2007) 8:138. doi: 10.1186/1471-2164-8-138, PMID: 17537256 PMC1899180

[39] Stewart P Shen C Zhao D Goldmann W. Genetic analysis of the SPRN gene in ruminants reveals polymorphisms in the alanine-rich segment of shadoo protein. JJ Gen Virol. (2009) 90:2575–2580. doi: 10.1099/vir.0.011494-0, PMID: 19515828

[40] Won SY Kim YC Kim SK Jeong BH. The first report of genetic and structural diversities in the sprn gene in the horse, an animal resistant to prion disease. Genes (Basel). (2019) 11:39. doi: 10.3390/genes11010039, PMID: 31905681 PMC7016944

[41] Sanchez-Garcia J Fernandez-Funez P. D159 and S167 are protective residues in the prion protein from dog and horse, two prion-resistant animals. Neurobiol Dis. (2018) 119:1–12. doi: 10.1016/j.nbd.2018.07.011, PMID: 30010001 PMC6139044

[42] Fassler JS Skuodas S Weeks DL Phillips BT. Protein aggregation and disaggregation in cells and development. J Mol Biol. (2021) 433:167215. doi: 10.1016/j.jmb.2021.167215, PMID: 34450138 PMC8530975

[43] Jumper J Evans R Pritzel A Green T Figurnov M Ronneberger O. Highly accurate protein structure prediction with AlphaFold. Nature. (2021) 596:583–9. doi: 10.1038/s41586-021-03819-2, PMID: 34265844 PMC8371605

